# Enhancing angiogenesis and inhibiting apoptosis: evaluating the therapeutic efficacy of bone marrow mesenchymal stem cell-derived exosomes in a DHEA-induced PCOS mouse model

**DOI:** 10.1186/s13048-024-01445-w

**Published:** 2024-06-05

**Authors:** Xiaojing Teng, Zhiyi Wang, Xiaolei Wang

**Affiliations:** 1 Department of Clinical Laboratory, Affiliated Hangzhou First People’s Hospital, School of Medicine, Westlake University, Hangzhou, Zhejiang China; 2 Department of Clinical Laboratory, Hangzhou Women’s Hospital (Hangzhou Maternity and Child Health Care Hospital), No. 369, Kunpeng Road, Shangcheng District, Hangzhou, Zhejiang 310008 China

**Keywords:** Bone marrow mesenchymal stem cells, Exosomes, Polycystic ovary syndrome, Ovarian morphology, Apoptosis, Therapeutic application

## Abstract

**Background:**

Polycystic Ovary Syndrome (PCOS) is a widespread endocrine disorder among women, characterized by symptoms like ovarian cysts, hormonal imbalance, and metabolic issues. This research evaluates the therapeutic potential of Bone Marrow Mesenchymal Stem Cell-derived exosomes (BMSC-Exo) in treating PCOS symptoms within a mouse model.

**Methods:**

BMSC-Exo were isolated from NMRI mice, characterized using Transmission Electron Microscopy (TEM) and Nanoparticle Tracking Analysis (NTA), and administered to a PCOS mouse model induced by dehydroepiandrosterone (DHEA). The efficacy of BMSC-Exo was assessed in three groups of mice: a control group, a PCOS group, and a PCOS group treated with intravenous BMSC-Exo. Morphological changes in ovarian tissue were examined by Hematoxylin and Eosin (H&E) staining, apoptosis was determined using the TUNEL assay, and CD31 expression was analyzed through immunofluorescent staining to assess angiogenic activity.

**Results:**

The existence of BMSCs-Exo was confirmed via TEM and NTA, revealing their distinct cup-shaped morphology and a size range of 30 to 150 nanometers. H&E staining revealed that BMSCs-Exo treatment improved ovarian morphology in PCOS models, increasing corpora lutea and revitalizing granulosa cell layers, suggesting a reversal of PCOS-induced damage. TUNEL assays showed that BMSCs-Exo treatment significantly reduced apoptosis in PCOS-affected ovarian cells to levels comparable with the control group, highlighting its role in mitigating PCOS-induced cellular apoptosis. Immunofluorescence for CD31 indicated that BMSCs-Exo treatment normalized endothelial marker expression and angiogenic activity in PCOS models, suggesting its effectiveness in modulating the vascular irregularities of PCOS. Collectively, these findings demonstrate the therapeutic potential of BMSCs-Exo in addressing ovarian dysfunction, cellular apoptosis, and aberrant angiogenesis associated with PCOS.

**Conclusion:**

The study substantiates the role of BMSC-Exo in mitigating the deleterious effects of PCOS on ovarian tissue, with implications for enhanced follicular development and reduced cellular stress. The modulation of CD31 by BMSC-Exo further highlights their potential in normalizing PCOS-induced vascular anomalies. These findings propel the need for clinical investigations to explore BMSC-Exo as a promising therapeutic avenue for PCOS management.

## Background

Polycystic Ovary Syndrome (PCOS) is a prevalent endocrine disorder, affecting about 10% of women in their reproductive years [1]. This syndrome is characterized by symptoms such as menstrual irregularities, pronounced hyperandrogenism, and polycystic ovarian morphology [2], leading to significant health challenges. Beyond its direct impact on reproduction, PCOS is linked to a range of complications including infertility, metabolic issues, and increased cardiovascular risk [3, 4]. The complex etiology of PCOS makes its comprehensive management challenging.

Current treatments for PCOS include lifestyle modifications, pharmacological interventions, and surgical options, each with inherent limitations. Lifestyle modifications, such as weight loss through diet and exercise, are often recommended as a first-line treatment. While these changes can significantly improve symptoms and reduce metabolic complications, they require sustained effort and may not be effective for all patients [5]. Pharmacological treatments, such as oral contraceptives, are commonly used to regulate menstrual cycles and reduce hyperandrogenism, but they do not address insulin resistance and may have side effects like increased risk of thromboembolism. Metformin, used to improve insulin sensitivity, is limited by gastrointestinal side effects and variable efficacy [6]. Anti-androgens, like spironolactone, can reduce symptoms of hyperandrogenism but are contraindicated in pregnancy and may have other side effects [7]. Surgical options, such as ovarian drilling, provide an alternative for patients unresponsive to medical treatments; however, these procedures are invasive and carry risks of complications and potential ovarian damage [8].

Recent research has brought to light the significant role of exosomes—small extracellular vesicles secreted by cells-in cellular communication. These vesicles, containing a variety of biomolecules like proteins, lipids, and nucleic acids, are crucial in various physiological and pathological processes [9, 10]. Their influence extends to diverse conditions, including cancer and neurodegenerative diseases [11, 12].

In reproductive biology, a novel link between exosomes and PCOS is emerging. Preliminary studies suggest the therapeutic promise of mesenchymal stem cell-derived exosomes in addressing PCOS-related issues [13, 14]. The intricate molecular interactions facilitated by exosomes may tackle key PCOS-related problems, including follicular atresia, insulin resistance, and inflammation [15]. This emerging connection between exosomes and PCOS presents a new avenue for understanding and treating the condition.

Our study investigates the therapeutic potential of BMSCs-Exo in mitigating the multifaceted pathophysiology of PCOS.

## Methods

### Isolation and profiling of BMSC-Derived exosomes

In this study, we isolated BMSCs-Exo from 6 to 8-week-old female NMRI mice. The femoral and tibial bones were the primary sites for marrow extraction. Following a modified protocol based on the work of Claudia et al [16], we used density gradient centrifugation to isolate Bone Marrow Mesenchymal Stem Cells (BMSCs). Only cells from the third to fifth passages, which had reached approximately 80% confluence, were utilized. These cells were then incubated in exosome-free medium for a period of 48 h.

The initial step in exosome isolation involved a low-speed centrifugation at 300 g for 10 min, which served to clear the sample of cellular debris. This was followed by a critical ultracentrifugation stage at 100,000 g for 70 min, aimed at concentrating and purifying the exosomes. Structural examination of the isolated exosomes was conducted using TEM, highlighting their typical bilayered appearance. NTA confirmed that the exosome size predominantly ranged between 30 and 150 nanometers.

### Establishment of PCOS mouse model and BMSCs-Exo intervention

In our investigation of the therapeutic potential of BMSCs-Exo in PCOS, we utilized a DHEA-induced PCOS model in female mice, as described by Smith et al. [17]. Female NMRI mice, aged between 6 and 8 weeks, were subjected to daily subcutaneous DHEA injections (6 mg/100 g body weight) in 0.1 ml of sesame oil for a period of 21 days. This protocol effectively induced PCOS-like symptoms, characterized by disrupted ovulatory patterns and hormonal imbalances. The 21-day period was chosen based on previous studies demonstrating its sufficiency to induce PCOS-like symptoms in mice models using DHEA. This timeframe allows for the development of disrupted ovulatory patterns and hormonal imbalances characteristic of PCOS, providing a reliable basis for evaluating therapeutic interventions [18]. The dosage of 6 mg/ (100 g.d) DHEA was chosen based on established protocols from previous studies that have effectively induced PCOS-like symptoms in animal models. This specific dosage is well-documented to reliably produce the hormonal and morphological characteristics of PCOS, such as hyperandrogenism, disrupted estrous cycles, and ovarian cyst formation​ [19, 20].

The experimental protocol included a total of 15 mice, which were divided into three distinct groups for a comprehensive analysis. In the Control group, mice were administered subcutaneous injections of 0.1 ml sesame oil as a placebo. For the PCOS group, the regimen involved subcutaneous injections of 6 mg/ (100 g.d) DHEA, supplemented with 0.1 ml sesame oil. In the BMSCs-Exo group, after initial treatment with DHEA, an intravenous dose of BMSCs-Exo suspension (100 µg/Kg) was administered on the 21st day. This dose was determined based on prior studies to ensure accuracy and consistency in the treatment methodology.

### Euthanasia procedure

The euthanasia of the mice was carried out by administering an overdose of anesthesia, specifically through an intraperitoneal injection of Sodium Pentobarbital at a concentration of 3%, administered at three times the standard dosage. Throughout the process, close observation was maintained for signs such as corneal reflex, muscle relaxation, and reaction to pain. Injection was halted once the desired anesthetic effect was achieved. We continuously monitored vital signs like blood pressure and heart rate. Euthanasia confirmation was based on the absence of thoracic movement, pallor of the eyelids, and a lack of response to visual stimuli, ensuring that the process was humane and ethical.

### Post-euthanasia procedures

After euthanasia, ovarian tissues were carefully extracted for further examination. Additionally, blood samples were collected from the orbital sinus, and the serum was isolated and stored at -80 °C for subsequent analysis. While heart puncture provides a larger volume of blood, orbital sinus sampling was chosen to minimize the stress and invasiveness for the mice.

### Ethical compliance

All experimental procedures involving live animals in this study were conducted in accordance with the ethical standards set forth by the Institutional Animal Care and Use Committee of Youshu Life Sciences (Approval ID: YS-m20231100). Our methodology adhered strictly to the ARRIVE guidelines, with a focus on minimizing animal distress, in alignment with the Guide for the Care and Use of Laboratory Animals.

### Analysis of ovarian tissue morphology using H&E staining

For morphological analysis of ovarian tissues, we utilized the H&E staining method. Ovarian tissues were harvested for histological examination 28 weeks post-treatment. The 28-week period was chosen to comprehensively evaluate the long-term effects of the treatment on PCOS symptoms and assess any potential chronic effects. Initially, the tissues were fixed in 4% formaldehyde, preserving cellular integrity. This was followed by a graded dehydration process using increasing concentrations of ethanol. After dehydration, tissues were embedded in paraffin for sectioning. The tissue samples were cut into 5 mm sections to ensure uniformity and facilitate histological analysis. The sections were then stained: Hematoxylin for blue-purple nuclear visualization and Eosin for pink cytoplasmic staining.

Under light microscopy, these stained sections were scrutinized to evaluate histopathological changes, with a particular emphasis on follicular count analysis. This allowed for an accurate assessment of the treatment’s impact on ovarian follicular dynamics.

### Identification of Apoptotic Nuclei in oocytes using the TUNEL assay

For detecting apoptotic nuclei within oocytes, we conducted the TUNEL assay, adhering to the protocol of Roche’s In Situ Cell Death Detection Kit. The process began with deparaffinization and rehydration of ovarian tissue sections. These sections were then subjected to Proteinase K enzymatic digestion to permeabilize the cells and expose DNA ends. Post-digestion, sections were treated with Terminal deoxynucleotidyl Transferase (TdT) enzyme to label fragmented DNA. After washing in PBS, sections were incubated with a fluorescently tagged antibody, enabling visualization of the labeled DNA fragments. The sections were stained with Diaminobenzidine (DAB), facilitating the identification of apoptotic cells under fluorescence microscopy.

Microscopic analysis focused on follicles, particularly assessing the presence of apoptotic cells. Follicles with over 10% TUNEL-positive granulosa cell nuclei were classified as having apoptotic cells, allowing for quantitative evaluation of apoptosis in the ovarian tissues.

### Endothelial marker CD31

Angiogenic processes were gauged by inspecting the luteal expression of CD31, an endothelial marker indicative of vascular development. The methodology included preparing paraffin-embedded ovarian tissue sections, which were then rehydrated through a graded series of alcohol after xylene treatment. Proteolytic digestion preceded a blockage phase for nonspecific interactions using hydrogen peroxide. The sections received dual PBS washes before a two-hour primary antibody incubation. This step was followed by a triple PBS rinse and an hour-long incubation with a conjugated secondary antibody. A trio of PBS washes cleared the sections, which were then finalized on slides for fluorescence microscopic examination. This analysis focused on quantifying CD31-positive cell prevalence to interpret angiogenic signaling within the tissue samples.

### Statistical analysis

For statistical evaluation, GraphPad Prism software version 8.0 (GraphPad Software, San Diego, CA, USA) was utilized. In this study, three distinct groups were analyzed: control, PCOS and BMSCs-Exo group, with 5 biological replicates per group, totaling 15 samples for the initial set. For the procedures involving BMSCs transfection and exosome isolation, three biological replicates were assessed to ensure the reliability of the exosome preparation process. Statistical comparisons across the groups were conducted using one-way Analysis of Variance (ANOVA), with Tukey’s post hoc test for pairwise comparisons to identify significant differences. *P*-values of < 0.05, < 0.01, and < 0.001 were considered statistically significant, guiding the interpretation of treatment effects and differences between the experimental groups. *P*-values of < 0.05 indicate significant differences, < 0.01 indicate highly significant differences, and < 0.001 indicate extremely significant differences.

## Results

### Characterization of BMSCs-Exo morphology and size distribution

**Fig. 1:**
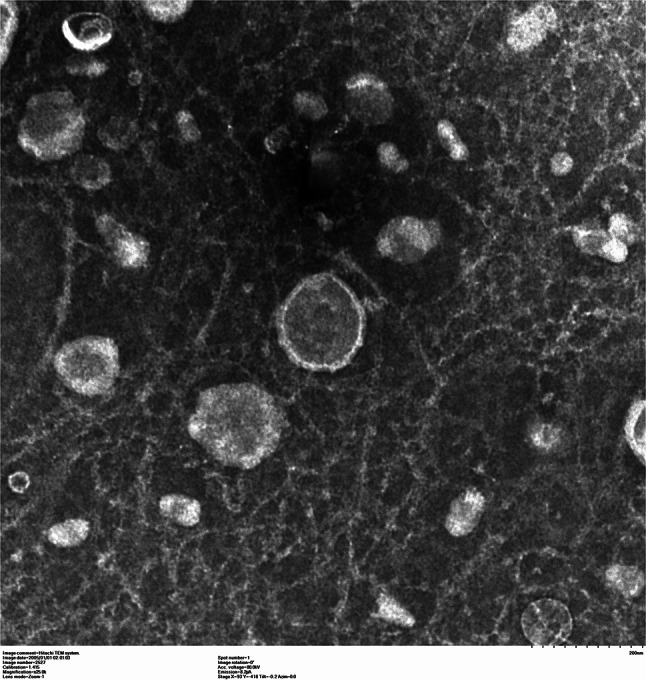
Exosome vesicles under transmission electron microscopy. This image showcases the distinctive cup-shaped morphology of exosome vesicles, crucial for cellular communication, highlighting the intricate vesicular surfaces vital for various biological functions. Scale bar = 200 nm

The structural properties and size metrics of BMSCs-Exo were delineated employing sophisticated microscopic methodologies. Transmission TEM revealed the ultrastructural details of BMSCs-Exo. As shown in Fig. 1, these exosomes exhibited a typical cup-shaped morphology with circular or ovoid shapes, encapsulating intraluminal contents. The TEM images distinctly displayed the characteristic bilayer membrane of BMSCs-Exo, marked by a pattern of dark and light bands, indicating their structural integrity.

**Fig. 2:**
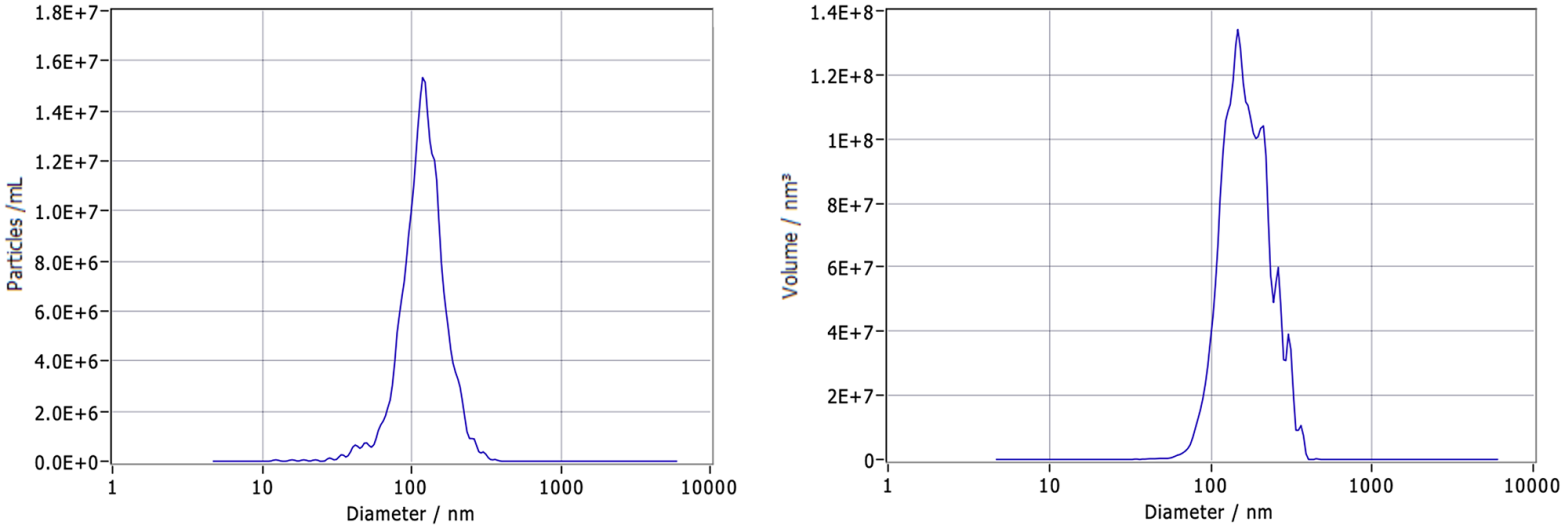
Exosome nanoparticle size distribution. This figure illustrates the size distribution of exosome nanoparticles, measured using nanoparticle tracking analysis (NTA), which shows a range from 30 to 150 nanometers

In terms of size distribution, BMSCs-Exo generally ranged from 30 to 150 nanometers. This size range was confirmed through NTA, which provided a quantitative assessment of their dimensions, as illustrated in Fig. 2. Variability in the size and shape of BMSCs-Exo was observed, potentially reflecting the diversity in their biological state and cellular origin.

### BMSCs-Exo alleviates ovarian tissue alterations in PCOS: H&E staining technique

In this study, ovarian tissue morphology was examined through H&E staining to identify variations under normal and PCOS-affected conditions. Ovarian samples from the control group exhibited standard histological features, characterized by a rich presence of intact corpora lutea, aligning with regular ovarian function. Conversely, the PCOS model presented a distinct shift in follicular structure, notably with an elevated occurrence of cystic follicles and a reduction in both antral follicles and corpora lutea. This pattern suggests a disruption in normal ovarian activities, possibly affecting ovulation.

**Fig. 3:**
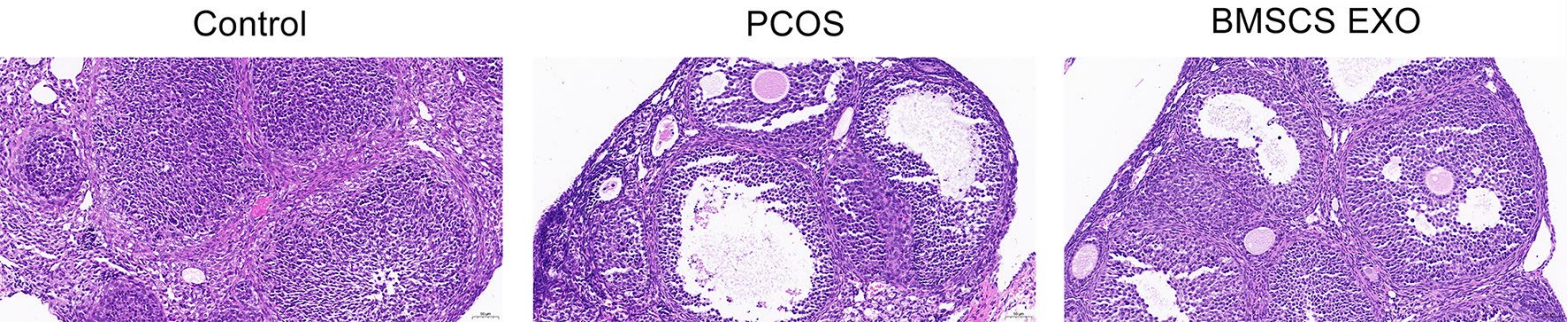
H&E staining outcomes in PCOS and treatment groups (*n* = 15). This figure demonstrates the impact of BMSCs-Exo treatment on ovarian tissue morphology. H&E staining highlights the abnormal ovarian architecture in the PCOS group, contrasting with the improved ovarian environment observed after BMSCs-Exo treatment, suggesting a potential role in restoring ovulatory function. Scale bar = 50 μm

Notably, ovarian tissues from the group receiving BMSCs-Exo therapy showed marked histological improvement. This group evidenced a significant increase in corpora lutea and a revitalization of the granulosa cell layers when compared to the PCOS group. Such morphological recovery in the BMSCs-Exo treated group indicates a reversal of PCOS-induced damage, supporting follicular development and enhancing luteal integrity. These observations, exemplified in Fig. 3, highlight the potential of BMSCs-Exo as a promising intervention for rectifying PCOS-associated ovarian dysfunctions.

### BMSCs-Exo reduces cell apoptosis in PCOS: TUNEL assay

The TUNEL assay, an established method for detecting cellular apoptosis, was utilized to identify DNA fragmentation in cells. In this study, ovarian cells from the control group showed a minimal presence of TUNEL-positive cells, indicative of a low rate of apoptosis, consistent with healthy cellular functioning. In stark contrast, ovarian cells from the PCOS group exhibited a marked increase in TUNEL-positive cells (*P* < 0.001). This observation suggests enhanced apoptotic processes, aligning with the known cellular stress and damage associated with PCOS.

**Fig. 4:**
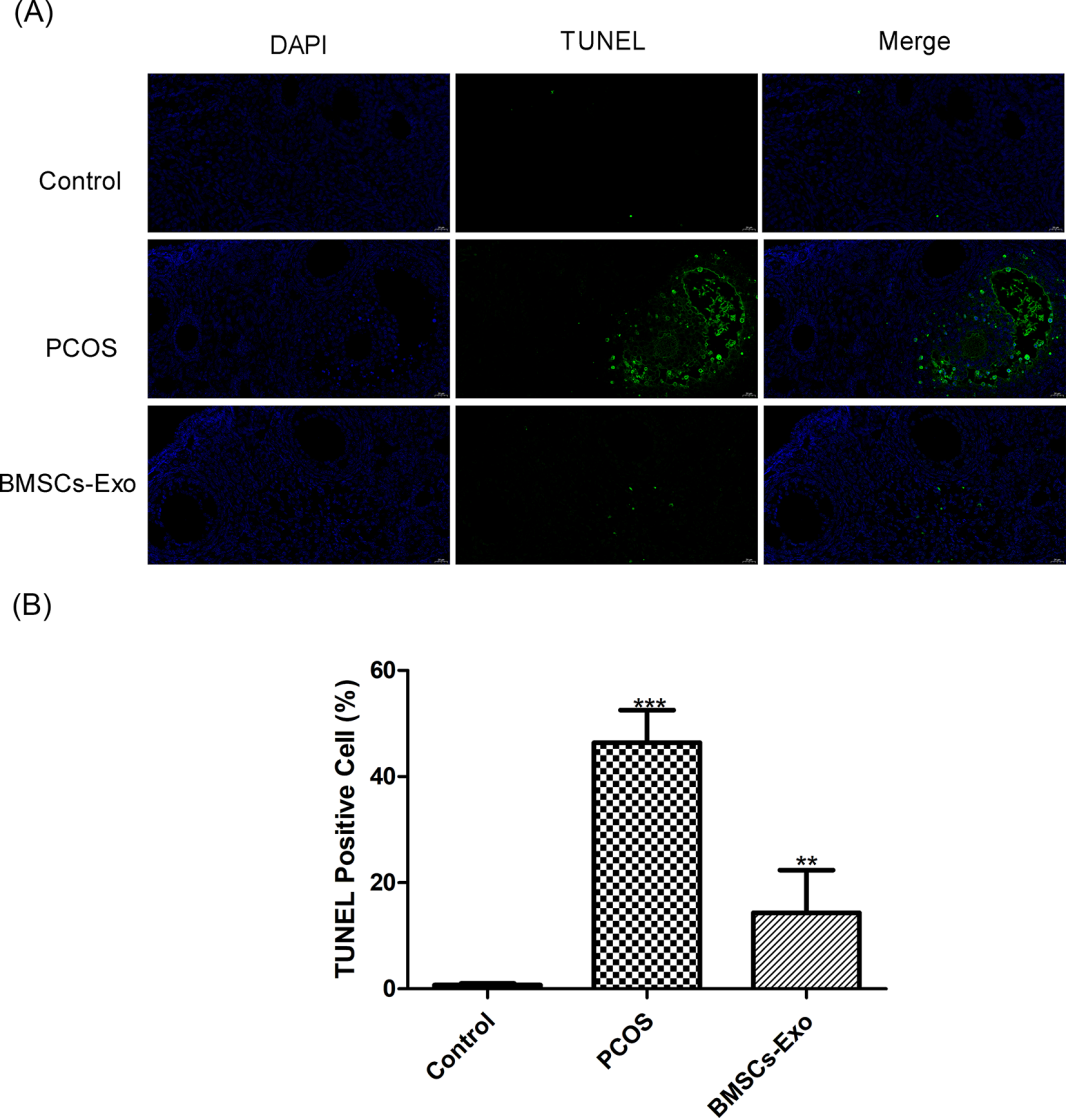
TUNEL apoptosis detection outcomes (*n* = 15). (**A**) TUNEL apoptosis fluorescence image. Scale bar = 20 μm. (**B**) TUNEL apoptosis cell column chart, *P < 0.05, **P < 0.01, ***P < 0.001 vs. Control group

Significantly, ovarian cells from mice treated with BMSCs-Exo displayed a considerable reduction in TUNEL-positive cells, closely mirroring the levels observed in the control group (*P* < 0.01). This notable decrease in apoptotic cell count in the BMSCs-Exo treated group suggests a mitigating effect of the treatment on cell apoptosis associated with PCOS. These findings, as illustrated in Fig. 4A and B, support the hypothesis that BMSCs-Exo treatment could be an effective therapeutic approach in reducing cellular apoptosis in PCOS, potentially offering a novel pathway for managing the syndrome.

### BMSCs-Exo modulates angiogenesis in PCOS: endothelial marker CD31

**Fig. 5:**
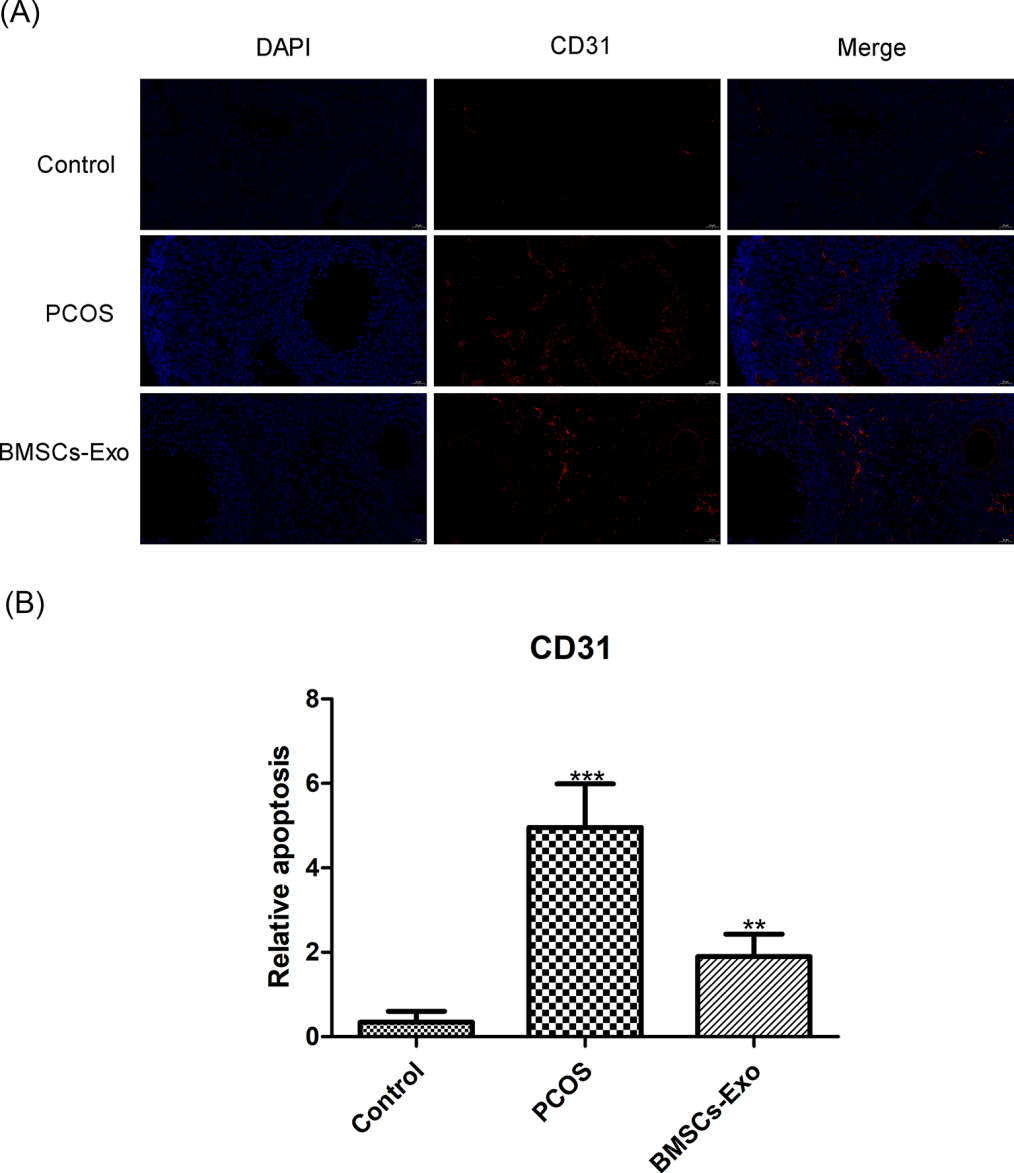
CD31 Expression Analysis in Murine Models (*n* = 15) (**A**) CD31 Fluorescence Imaging. Scale bar = 20 μm (**B**) CD31 Expression Variance Chart, *P < 0.05, **P < 0.01, ***P < 0.001 vs. Control group

Immunofluorescence assays were utilized to assess CD31, a key endothelial marker, across different groups in this investigation. Analysis of control specimens revealed CD31 expression at expected physiological levels, indicative of normal endothelial function and angiogenic processes. In contrast, ovarian tissues from the PCOS cohort displayed significant elevation in CD31 expression, which is indicative of increased endothelial activation and angiogenesis (*P* < 0.001). These changes may be associated with the underlying inflammatory processes and vascular dysfunctions inherent in PCOS. Remarkably, subsequent to BMSCs-Exo application, a discernible reduction in CD31 expression was observed, highlighting the modulatory effect of BMSCs-Exo on endothelial markers (*P* < 0.01). This downregulation suggests a therapeutic potential for BMSCs-Exo in attenuating the heightened angiogenic activity observed in PCOS. The data suggest that BMSCs-Exo may play a role in normalizing the aberrant vascular responses, providing a new avenue for therapeutic intervention in PCOS. The implications of these findings are substantial, offering insights into the modulation of angiogenesis by BMSCs-Exo in the context of PCOS pathophysiology (Fig. 5A and B).

## Discussion

The study enhances current understanding of PCOS, a prevalent endocrine disorder impacting a significant number of women in their reproductive years. By investigating the application of BMSCs-Exo, the research targets the urgent need for innovative therapeutic strategies to address the complex symptoms of PCOS, including hormonal imbalances and the development of polycystic ovaries [21]. Despite extensive research, the intricate etiology and multifaceted pathophysiology of PCOS continue to present substantial therapeutic challenges. In this context, the study on BMSCs-Exo provides new perspectives, aligning with ongoing research efforts [22, 23].

BMSCs-Exo, as carriers of diverse bioactive molecules, play a pivotal role in facilitating intercellular communication, which is particularly crucial in managing PCOS. The findings demonstrate the effectiveness of BMSCs-Exo in correcting both morphological and cellular abnormalities frequently observed in PCOS. This aligns with the existing body of research, including the work of Dr. Hang-Soo and others, which underscores the importance of exosomes in modulating cellular behavior in the context of PCOS [24]. Significantly, this study goes beyond current research by providing novel insights into the multifaceted therapeutic effects of BMSCs-Exo in a PCOS model. Particularly noteworthy is the ability of BMSCs-Exo to modulate angiogenesis through CD31 expression, offering a new understanding of PCOS-related vascular anomalies. These findings contribute to the growing body of knowledge on the potential of BMSCs-Exo in reproductive health, underscoring their role not just in cellular communication but also as agents capable of reversing key PCOS pathological features.

Recent research has underscored the potential of exosomes, especially those derived from BMSCs, as effective therapeutic agents in PCOS management. In this study, mesenchymal stem cell exosomes were extracted from mouse bone marrow, with a focus on their crucial role as intercellular communicators carrying bioactive molecules that influence the behavior of recipient cells [25–27]. Through meticulous characterization via Transmission TEM and NTA, the exosomes’ size and morphology were confirmed, aligning with established exosomal profiles [28]. This verification is essential, underpinning the therapeutic implications of the findings within the broader scope of PCOS treatment.

In this study, H&E staining was employed to highlight morphological changes in ovarian tissue characteristic of PCOS, emphasizing its importance in identifying cellular and structural alterations such as cystic follicles and granulosa cell layer modifications. The application of this staining technique revealed that treatment with BMSCs-Exo mitigated these PCOS-induced aberrations. This finding is particularly noteworthy as it extends the current understanding established by researchers like Dr. Zhang and colleagues. Their work on a rat model of PCOS demonstrated similar restorative effects of BMSCs-Exo on follicular maturation and cystic structures [29].

Apoptosis, a critical cellular aspect in PCOS, exhibited a notable increase in the PCOS model used in this study, as evidenced by the TUNEL assay [30, 31]. Importantly, the research demonstrated a significant decrease in apoptotic levels following BMSCs-Exo intervention. This aspect of the study is distinctive, not only aligning with the anti-apoptotic observations reported in previous research [32, 33], but also providing deeper insights into the specific role and mechanisms of BMSCs-Exo in a PCOS context. The distinct contribution of this study lies in its in-depth examination of how BMSCs-Exo can effectively alter apoptotic processes in PCOS, thereby offering novel insights into potential therapeutic approaches for the disorder. This novel perspective distinguishes the study within the broader field of PCOS research.

Building on these insights, the study also delves into another critical aspect of PCOS pathology – angiogenesis. The revelation of how BMSCs-Exo treatment modulates CD31 expression brings forth a pivotal understanding of PCOS-related angiogenesis. This finding is crucial, as CD31 is a key marker for vascular dynamics in PCOS [34]. The research not only corroborates existing literature on exosomes’ regulatory roles in cellular signaling [35] but also distinctively highlights their specific impact on angiogenic processes within PCOS [36]. By demonstrating how BMSCs-Exo can target and potentially reverse abnormal angiogenic patterns indicated by CD31 expression, the study offers novel insight into PCOS pathophysiology. This focus on a particular molecular pathway suggests that BMSCs-Exo could provide a more targeted and effective therapeutic approach, addressing the critical role of angiogenesis in the progression of PCOS symptoms. Consequently, these findings enrich the current understanding of exosomal therapy in PCOS, proposing BMSCs-Exo as a refined strategy for managing the disorder’s vascular complexities.

The current investigation into BMSCs-Exo as a therapeutic intervention for PCOS is notable for its in-depth analysis of the cellular and molecular responses to treatment. Employing advanced techniques like TEM, NTA, and TUNEL assays, the study offers a detailed understanding of how BMSCs-Exo can modulate critical factors like angiogenesis, as evidenced by CD31 expression, and apoptosis in PCOS. These findings are significant as they contribute new perspectives to the potential of exosomes in targeted therapy for PCOS, a complex and multifaceted disorder.

However, the study is not without limitations. The use of a specific animal model may limit the direct applicability of the results to human PCOS patients. Additionally, the small sample size might constrain the statistical power and generalizability of the conclusions. Future research could aim to validate these promising findings in more diverse and larger populations and perhaps explore the potential long-term effects and clinical applications of BMSCs-Exo treatment in PCOS.

In conclusion, this study marks a substantial advancement in the field of PCOS research. It highlights the therapeutic promise of BMSCs-Exo in managing key pathological features of the syndrome, such as altered angiogenesis and increased apoptosis. By delineating the effects of BMSCs-Exo at a cellular level, the research opens new avenues for targeted treatments, moving towards more effective and personalized approaches in managing PCOS. The insights gained here lay a valuable foundation for ongoing and future studies aimed at combatting this prevalent endocrine disorder.

## Conclusion

In summary, this research presents significant advancements in the treatment of PCOS through the application of BMSCs-Exo. The study’s findings reveal the potential of BMSCs-Exo to modulate key aspects of PCOS pathology, notably in angiogenesis and apoptosis. These insights not only enhance our understanding of PCOS at the molecular level but also open new avenues for targeted therapeutic strategies. Ultimately, this research lays a foundation for future investigations into personalized treatments for PCOS, potentially improving outcomes for a significant number of affected women.

## Data Availability

All data generated or analyzed during this study are included in this published article and its supplementary information files.

## Abbreviations

PCOS: Polycystic Ovary Syndrome
BMSCs: Bone Marrow Mesenchymal Stem Cells
BMSCs-Exo: Bone Marrow Mesenchymal Stem Cell-derived exosomes
DHEA: Dehydroepiandrosterone
H&E: Hematoxylin and Eosin staining
TEM: Electron microscopy
NTA: Nanoparticle tracking analyzer
TdT: Terminal deoxynucleotidyl Transferase
DAB: Diaminobenzidine
ANOVA: Analysis of Variance
